# Exogenous Application of Amino Acids Alleviates Toxicity in Two Chinese Cabbage Cultivars by Modulating Cadmium Distribution and Reducing Its Translocation

**DOI:** 10.3390/ijms25158478

**Published:** 2024-08-03

**Authors:** Longcheng Li, Qing Chen, Shihao Cui, Muhammad Ishfaq, Lin Zhou, Xue Zhou, Yanli Liu, Yutao Peng, Yifa Yu, Wenliang Wu

**Affiliations:** 1 Beijing Key Laboratory of Biodiversity and Organic Farming, College of Resources and Environmental Sciences, China Agricultural University, Beijing 100193, China; lilongchengwy@163.com; 2 Beijing Key Laboratory of Farmland Soil Pollution Prevention and Remediation, College of Resources and Environmental Sciences, China Agricultural University, Beijing 100193, China; qchen@cau.edu.cn (Q.C.); shihaoc@foxmail.com (S.C.); zhoulin@cau.edu.cn (L.Z.); xuezhou0207@163.com (X.Z.); lyl_990824@163.com (Y.L.); 3 College of Life Sciences and Oceanography, Shenzhen University, Shenzhen 518060, China; ashfaqkamoka6@gmail.com; 4 School of Agriculture, Sun Yat-sen University, Shenzhen 523758, China; ytaopeng@mail.sysu.edu.cn; 5 College of Plant Protection, Yunnan Agricultural University, Kunming 650201, China; yuyf@harworld.com

**Keywords:** amino acids, cadmium translocation, Chinese cabbage, chemical forms, subcellular distribution

## Abstract

Plants communicate underground by secreting multiple amino acids (AAs) through their roots, triggering defense mechanisms against cadmium (Cd) stress. However, the specific roles of the individual AAs in Cd translocation and detoxification remain unclear. This study investigated how exogenous AAs influence Cd movement from the roots to the shoots in Cd-resistant and Cd-sensitive Chinese cabbage cultivars (Jingcui 60 and 16-7 cultivars). The results showed that methionine (Met) and cysteine (Cys) reduced Cd concentrations in the shoots of Jingcui 60 by approximately 44% and 52%, and in 16-7 by approximately 43% and 32%, respectively, compared to plants treated with Cd alone. However, threonine (Thr) and aspartic acid (Asp) did not show similar effects. Subcellular Cd distribution analysis revealed that AA supplementation increased Cd uptake in the roots, with Jingcui 60 preferentially storing more Cd in the cell wall, whereas the 16-7 cultivar exhibited higher Cd concentrations in the organelles. Moreover, Met and Cys promoted the formation of Cd-phosphate in the roots of Jingcui 60 and Cd-oxalate in the 16-7 cultivar, respectively. Further analysis showed that exogenous Cys inhibited Cd transport to the xylem by downregulating the expression of *HMA2* in the roots of both cultivars, and *HMA4* in the 16-7 cultivar. These findings provide insights into the influence of exogenous AAs on Cd partitioning and detoxification in Chinese cabbage plants.

## 1. Introduction

Cadmium (Cd) pollution poses a significant challenge to agricultural production in China [1,2]. The high toxicity and mobility of Cd in the plant systems has resulted in excessive Cd accumulation in plants [3,4]. Chinese cabbage is a widely cultivated crop in China, accounting for 15% of the total cultivated area and contributing 19% to the total yield of all vegetable crops [5]. The consumption of leafy vegetables, which is the primary exposure pathway, accounts for over 70–90% of Cd intake by humans [6]. Hence, reducing Cd accumulation in the edible parts of vegetables is essential for ensuring food security and human health.

The mechanism of Cd tolerance involves the sequestration of Cd in the cell wall to prevent its entry into the cell [7,8]. Endodermal suberization regulates the Cd content in the apoplastic pathway, affecting the transport of Cd to the aboveground parts [9,10]. Excess Cd is transported into vacuoles, which reduces the concentration of free Cd ions and protecting organelles [11]. Vacuoles play a decisive role in Cd detoxification through tonoplast transporters [12]. The ATP-binding cassettes (ABCs) *AtABCC1* and *AtABCC2* are responsible for transporting Cd-phytochelatin (PC) complexes to vacuolar storage [13]. ATPase (HMA), *HMA3*, is mainly expressed in the root tonoplast and is involved in the vacuolar storage of Cd [14]. Additionally, iron-regulated transporters (*IRT*s), *IRT1* and *IRT2*, which are close homologs of the Zrt/Irt-like protein (*ZIP*) family, encode high-affinity iron transporters [15]. Exogenous abscisic acid decreases Cd accumulation in *Arabidopsis* by inhibiting *IRT1*-mediated Cd uptake [16]. Multiple studies have reported that *HMA2* and *HMA4* regulate the loading of Zn and Cd in the xylem for long-distance transportation [17,18,19]. In *Arabidopsis*, *MYB43* negatively regulates Cd tolerance by inhibiting the transcription of *HMA2* and *HMA4* [20]. Foliar application of gibberellin significantly reduced the relative gene expression of *HMA2* and *HMA4*, thereby inhibiting Cd transport in lettuce [21]. Moreover, the chemical speciation of Cd affects its activity and mobility in plant tissues [22].

Supplying nutrients such as iron [23], sulfide [24,25], and selenium [26] can influence Cd uptake and translocation in plants by altering the subcellular distribution and chemical speciation of Cd. Cd toxicity can be alleviated at least partly by root exudates, which are involved in the complex roles of a wide array of amino acids (AAs) in the rhizosphere [27,28]. Exogenous AAs have diverse functions in Cd-stressed plants. For example, the application of glutamate (Glu), glycine (Gly), and cysteine (Cys) attenuates reactive oxygen species in rice [29]. The addition of Gly alters the concentrations of Cd in the apoplastic and symplastic pathways, thereby increasing Cd concentration in the wheat shoots [30]. Exogenous feeding of proline and histidine increased Cd influxes in the roots of *Solanum nigrum* and *S*. *torvum* but did not markedly increase the Cd leaf:root ratios [31]. In rice, the downregulation of Cd-related transporters, such as *NRAMPs*, *IRTs*, and *HMAs*, can reduce Cd uptake and facilitating Cd detoxification by adding Glu [32]. However, the mechanisms underlying the beneficial effects of AAs on Cd toxicity and translocation in leafy vegetables are poorly understood. We hypothesized that the exogenous application of AAs could modulate the subcellular distribution and chemical speciation of Cd and affect its uptake and translocation in Chinese cabbage. Our objectives were to (1) examine the effect of exogenous AAs on Cd accumulation in the shoot and root tissues of Chinese cabbage, (2) measure the impacts of different AAs on the subcellular distribution and chemical speciation of Cd, and (3) reveal the potential impact of various AAs on Cd concentration in the xylem and the relative expression of metal transport genes in the roots of both cultivars. Our study design allowed us to identify the potential of different AAs for reducing Cd toxicity and lowering the Cd concentration in the edible parts of leafy vegetables.

## 2. Results

### 2.1. Growth and Photosynthetic Activity

**Figure 1:**
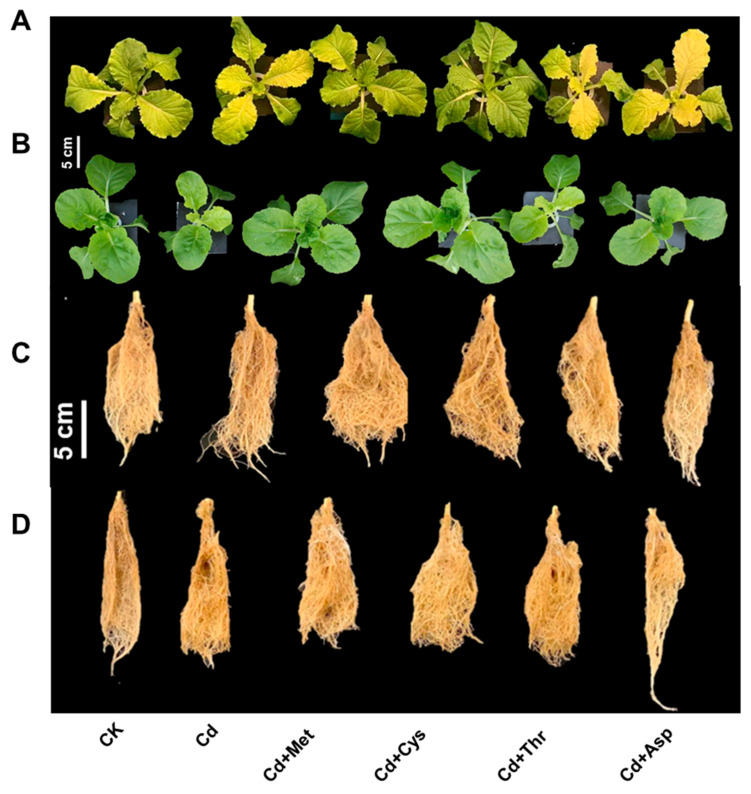
Effect of Met, Cys, Thr, and Asp on Chinese cabbage (Jingcui 60 and 16-7) growth (**A**,**B**) and root length (**C**,**D**).

**Figure 2:**
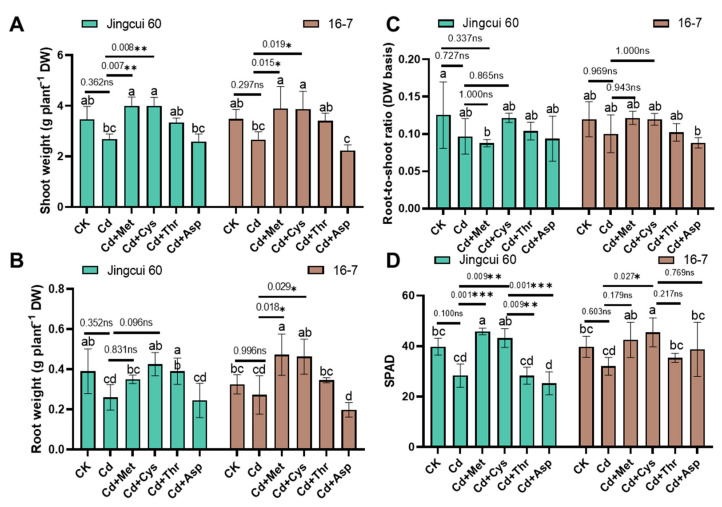
Effect of Met, Cys, Thr, and Asp on Chinese cabbage (Jingcui 60 and 16-7) shoot weight (g plant^−1^ DW) (**A**), root weight (g plant^−1^ DW) (**B**), root-to-shoot ratio (**C**), and soil plant analysis development (SPAD) values (**D**) under 5 μM Cd stress. Each value represents the mean ± SD (n = 3). Letters a–c indicate significant differences between treatments at *p* < 0.05. ANOVA with Tukey’s post hoc test used for the parametric analysis. Asterisks indicate significant differences between two groups: * *p* < 0.05; ** *p* < 0.01; *** *p* < 0.001. ns indicates no significant difference.

In the first experiment, we explored whether exogenous AAs are beneficial for Chinese cabbage under 5 μM Cd stress. The addition of AAs increased the growth of Chinese cabbage (Figure 1A–D and Figure 2A–C) and improved photosynthesis (Figure 2D). Specifically, Cd treatment alone decreased shoot DW by 23% compared to the control (CK), whereas Cd+(methionine) Met and Cd+(cysteine) Cys treatments resulted in a 12% and 11% increase in shoot DW, respectively (Figure 2A). Moreover, the application of Met and Cys significantly affected root biomass, especially in the 16-7 cultivar, which increased by 73% and 70%, respectively, compared to Cd alone (Figure 2B). Under Cd stress, both cultivars exhibited chlorosis in their recently developed leaves, as shown in Figure 1A,B. Supplementation with Met and Cys increased chlorophyll levels (as indicated by the SPAD value) by 37% and 63% in Jingcui 60 and by 60% and 63% in 16-7, respectively, compared with the sole Cd application (Figure 2D, *p* < 0.05). In contrast, such an increase in chlorophyll levels was not observed in the threonine (Thr) and aspartic acid (Asp) treatments.

### 2.2. Oxidative Damage in Chinese Cabbage

To validate whether exogenous AAs improve Cd resistance in Chinese cabbage, we investigated their protective effects on (malonaldehyde) MDA and H_2_O_2_ levels and the activity of antioxidant enzymes in both cultivars exposed to Cd stress. Cd stress significantly increased MDA and H_2_O_2_ levels in the shoots of both cultivars compared to those following CK treatment (Figure A4). Nevertheless, supplementation with Met, Cys, and Thr significantly reduced MDA and H_2_O_2_ levels in both cultivars compared to plants treated with Cd alone (Figure A4A,B). Met and Cys supplementation also notably enhanced the activities of CAT, POD, and SOD in both cultivars (Figure A4C–E).

### 2.3. Cd Uptake and Translocation

**Figure 3:**
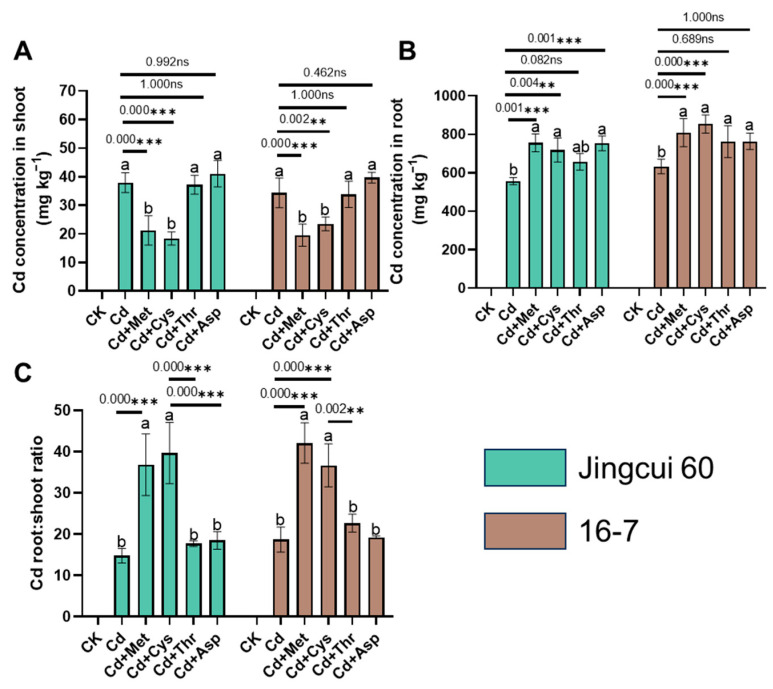
Effects of Met, Cys, Thr, and Asp applications on the Cd concentration (**A**,**B**) and the root:shoot ratio (**C**) in two Chinese cabbage cultivars under 5 μM Cd stress. Each value is the mean ± SD (n = 3). Letters a-c indicate significant differences between treatments at *p* < 0.05. ANOVA with Tukey’s post hoc test was used for the parametric analysis. Asterisks indicate significant differences between two groups: ** *p* < 0.01; *** *p* < 0.001. ns indicates no significant difference.

We determined the Cd concentration in Chinese cabbage and found that the supplementation of Met and Cys decreased the Cd concentration in the shoots by 44% and 52% in Jingcui 60 and by 43% and 32% in the 16-7 cultivar, respectively, compared with the sole Cd application (Figure 3A). In contrast, AA addition significantly increased the Cd concentration in the roots by 36%, 29%, 18%, and 35% in Jingcui 60 and by 28%, 35%, 21%, and 21% in the 16-7 cultivar (Figure 3B). We further calculated the root–leaf ratio of Cd in these treatments and found that Met and Cys significantly increased the Cd root–leaf ratio, suggesting that Met and Cys alleviated Cd toxicity, likely by reducing Cd translocation to the shoot and promoting Cd fixation in the root tissues (Figure 3C).

### 2.4. Cd Subcellular Distribution

**Figure 4:**
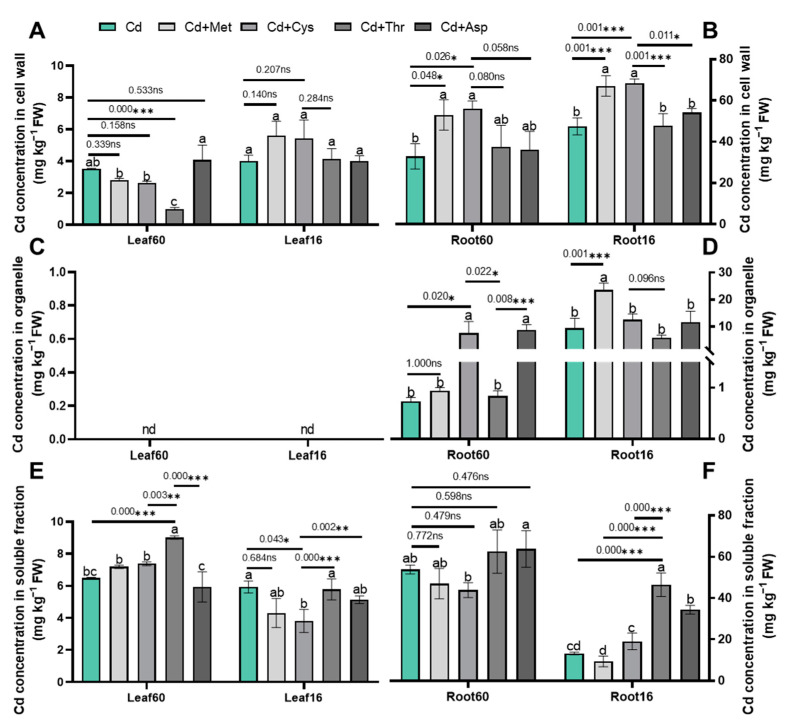
Effect of Met, Cys, Thr, and Asp applications on the subcellular distribution of Cd in the shoot (**A**,**C**,**E**) and root (**B**,**D**,**F**). “nd” indicates "not detected". Leaf60 refers to the leaf of the Jingcui 60 cultivar, Leaf16 refers to the leaf of the 16-7 cultivar, Root60 refers to the root of the Jingcui 60, and Root16 refers to the root of the 16-7 cultivar. Plant cells were separated into cell wall fraction (Fcw), organelle fraction (Fo), and soluble fraction (Fs), where n = 3. Letters a-c indicate significant differences between treatments at *p* < 0.05. ANOVA with Tukey’s post hoc test was used for the parametric analysis. Asterisks indicate significant differences between two groups: * *p* < 0.05; ** *p* < 0.01; *** *p* < 0.001. ns indicates no significant difference.

**Figure 5:**
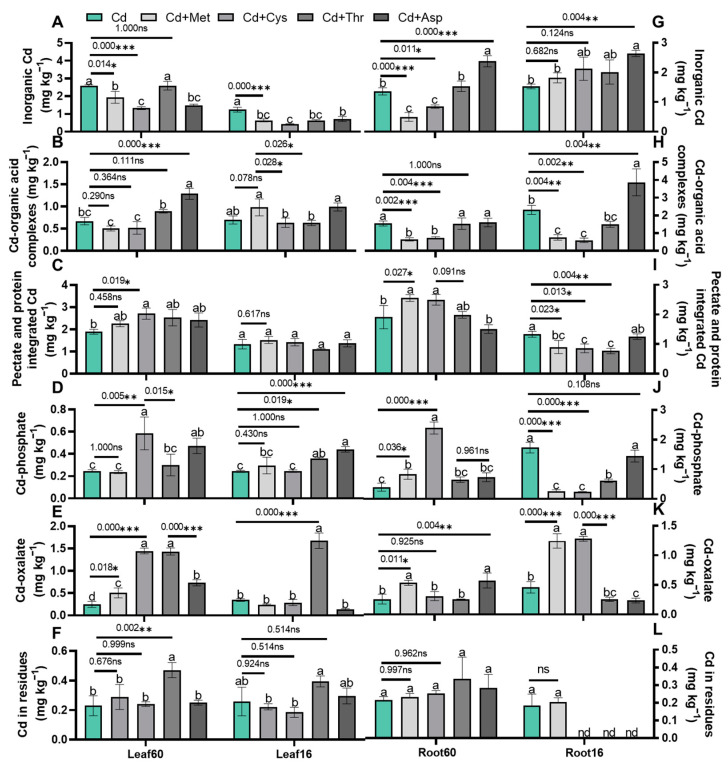
Concentrations of different chemical forms of Cd in the shoots (**A**–**F**) and roots (**G**–**L**) of Jingcui 60 and 16-7 cultivars with AA application. Leaf60 refers to the leaf of the Jingcui 60 cultivar, Leaf16 refers to the leaf of the 16-7 cultivar, Root60 refers to the root of the Jingcui 60, and Root16 refers to the root of the 16-7 cultivar. These chemical forms represent the fractions extracted by 80% ethanol (inorganic Cd, F1), deionized water (Cd-organic acid complexes, F2), 1 M NaCl (pectate-integrated Cd, F3), 2% acetic acid (Cd-phosphate, F4), 0.6 M HCl (Cd-oxalate, F5), and the residue (F6). Letters a-c indicate significant differences between treatments at *p* < 0.05. ANOVA with Tukey’s post hoc test was used for the parametric analysis. Asterisks indicate significant differences between two groups: * *p* < 0.05; ** *p* < 0.01; *** *p* < 0.001. ns indicates no significant difference.

**Figure 6:**
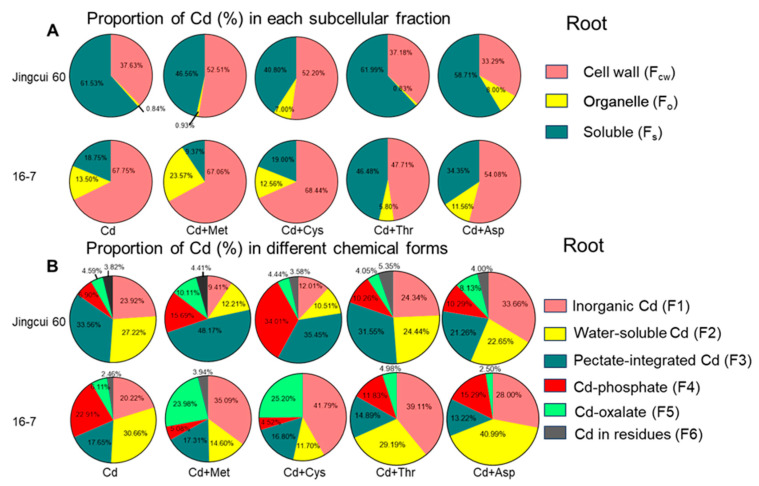
Proportion of Cd (%) in different subcellular fractions (**A**) and chemical forms of Cd (**B**) in the roots of both Chinese cabbage cultivars.

Given that the subcellular redistribution and chemical speciation of Cd are essential for improving Cd tolerance in plants, we hypothesized that exogenous AAs might improve Cd tolerance by modifying the subcellular distribution and chemical speciation of Cd in Chinese cabbage. To test this hypothesis, we determined the Cd concentrations in the cell walls, organelles, and soluble fractions, as well as in the six chemical forms present in the leaves and roots of both cultivars (Figure 4, Figure 5 and Figure 6). The distribution of Cd in the subcellular fractions of the leaves showed that exogenous Thr significantly decreased the Cd concentration in the cell wall fraction by 72% and increased the concentration in the soluble fraction by 38% in Jingcui 60 (Figure 4A,E, *p* < 0.05). In contrast, it did not significantly affect the Cd + Met, Cd + Cys, or Cd + Asp treatments relative to the plants treated only with Cd (Figure 4A,C,E). The addition of Cys decreased the Cd concentration in the soluble fraction of the leaves of the 16-7 cultivar (Figure 4E, *p* < 0.05). These results indicate that the addition of AAs alters Cd allocation in the subcellular fraction of leaves, and this effect depends on the type of AAs involved.

Next, we analyzed the Cd concentration and average proportion of Cd in the subcellular fractions of the roots (Figure 4B,D,F). Supplementation with Met and Cys notably elevated the Cd concentration in the cell wall fraction of both cultivars, whereas this pattern was not observed with Thr or Asp supplementation (Figure 4B, *p* < 0.05). In the organelle fraction, the Cd concentration was significantly increased by the Cd + Cys and Cd + Asp treatments of Jingcui 60 and by the Cd + Met treatment of 16-7 cultivar (Figure 4D, *p* < 0.05). We found that Cd + Thr and Cd + Asp treatments increased the Cd concentration in the soluble fraction of the 16-7 cultivar; however, no such increase was observed in Jingcui 60 (Figure 4F, *p* < 0.05).

**Figure 7:**
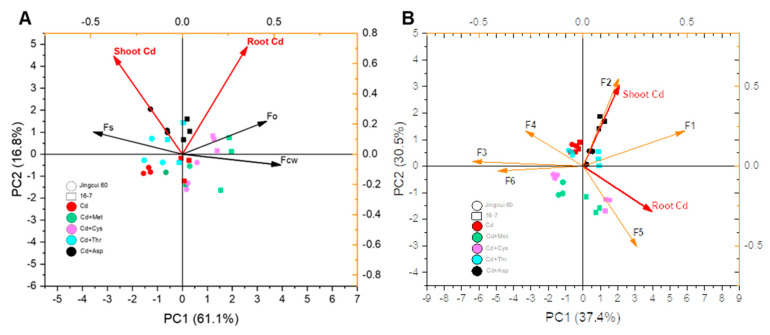
Principal component analysis (PCA) was used to examine the relationship between the subcellular distribution and chemical forms of Cd in the root, and Cd concentrations in the shoot and root (**A**,**B**). The circle and square refer to the samples from the Jingcui 60 and 16-7 cultivars, respectively. The colors (red, green, pink, blue, and black, respectively) represent Cd, Cd + Met, Cd + Cys, Cd + Thr, and Cd + Asp treatments. Abbreviations: shoot Cd, Cd concentration in the shoots; DW, dry weight; Fcw, cell wall fraction; Fo, organelle fraction; Fs, soluble fraction; F1, inorganic Cd extracted by 80% ethanol; F2, Cd-organic acid complexes extracted by DI water; F3, pectate-integrated Cd; F4, insoluble Cd-phosphate; F5, Cd-oxalate; F6, Cd in the residues.

Moreover, we assessed the average proportion of Cd in the subcellular distribution, revealing that Cd was predominantly localized in the cell wall fraction, accounting for 33–68% of the total (Figure 6A). On average, the cell wall fraction contained 43% of the total Cd in Jingcui 60 and 61% in the 16-7 cultivar (Figure 6A). The soluble fraction was 54% for Jingcui 60 and 26% for 16-7, and the organelle fraction was 4% for Jingcui 60 and 13% for 16-7 (Figure 6A). The results of the principal component analysis (PCA) demonstrate a positive correlation between Cd concentration in the shoots and the root soluble fractions of both varieties, which was contrasted by a discernible negative association with Cd in the cell wall and organelle fractions (Figure 7A). In the cell wall fraction, the order for Jingcui 60 was Cd + Met (53%) > Cd + Cys (52%) > Cd (38)% > Cd + Thr (37%) > Cd + Met (33%), and for the 16-7 cultivar, it was Cd + Cys (68%) = Cd (68%) > Cd + Met (67%) > Cd + Asp (54%) > Cd + Thr (47%) (Figure 6A). These results indicate that the addition of AAs alters the distribution of Cd in the subcellular fractions, particularly in the cell wall fraction, and there are differences between the two cultivars.

### 2.5. Chemical Forms of Cd

To identify the key chemical species of Cd associated with its low translocation to shoots under exogenous AA application, we measured the concentrations of various chemical Cd species in the shoots and roots of Chinese cabbage (Figure 5). Among the chemical species, the predominant forms were pectate-bound Cd, constituting 34% of the roots of the Jingcui 60 cultivar, and Cd-organic acid complexes, comprising 31% of the roots of the 16-7 cultivar (Figure 5B). Importantly, PCA indicated a positive correlation between Cd concentration in the shoots of both cultivars and the levels of inorganic and Cd-organic acid complexes species in the roots (Figure 7B). Conversely, negative correlations were observed between the levels of pectate-integrated, phosphate, oxalate, and residual Cd speciation (Figure 7B). Treatments with Cd + Met and Cd + Cys significantly increased the proportion of Cd bound to phosphate in the roots of the Jingcui 60 cultivar and oxalate in the 16-7 cultivar, respectively, compared to plants treated with Cd alone (Figure 6B). Conversely, the proportions of inorganic and Cd-organic acid complexes were significantly higher in the Cd + Thr and Cd + Asp treatments than in the Cd + Met and Cd + Cys treatments (Figure 6B). These findings suggest that the binding of Cd to phosphate and oxalate plays a crucial role in the translocation and detoxification of Cd in Chinese cabbage supplemented with various AAs.

### 2.6. Cd Concentration in the Xylem and Relative Expression of Metal Transport Genes

**Figure 8:**
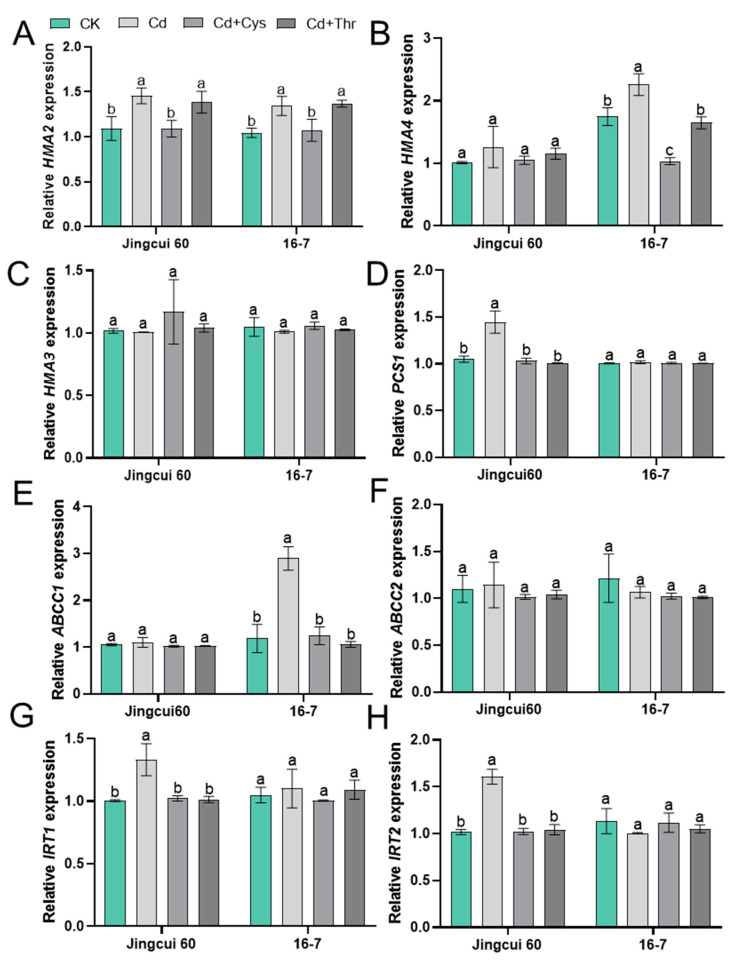
The expressions of *HMA2*, *HMA4*, *HMA3*, *PCS1*, *ABCC1*, *ABCC2*, *IRT1*, and *IRT2* in roots of both cultivars under Cd + Cys and Cd + Thr treatments (**A**–**H**). Data are presented as means ± SD, and different letters on the bars indicate significant differences at *p* < 0.05 (n = 3 plants for each replicate). ANOVA with Tukey’s post hoc test was used for the parametric analysis.

To experimentally validate the essential role of exogenous AAs in reducing Cd translocation to the shoot, we analyzed the Cd concentration in the xylem (Figure A5) and the expression of *HMA2*, *HMA4*, *HMA3*, *PCS1*, *ABCC1*, *ABCC2*, *IRT1*, and *IRT2* in the roots of the two cultivars under the Cd + Cys and Cd + Thr treatments (Figure 8A–H). Supplementation with Cys significantly decreased the Cd concentration in xylem sap by 48.28% and 50.30%, compared to Cd application alone, whereas there was no significant difference with Thr addition (Figure A5). The expression of *HMA2* was upregulated in both cultivars under Cd stress, whereas Cys addition significantly downregulated the expression of *HMA2* in both cultivars (Figure 8A). The expression of *HMA4* was downregulated by Cys and Thr applications in the 16-7 cultivar (Figure 8B). The expression of *PCS1*, *IRT1*, and *IRT2* was upregulated in the Jingcui 60 cultivar under Cd stress, while it did not significantly change in the 16-7 cultivar (Figure 8D,G,H). The application of Cys and Thr downregulated the expressions of *PCS1*, *IRT1*, and *IRT2* in the Jingcui 60 cultivar under Cd stress (Figure 8D,G,H). These results suggest that the response of relevant genes to Cd toxicity and exogenous AAs varies between the two cultivars and that exogenous AAs inhibit Cd transport to the xylem by regulating the expression of *HMA2* and *HMA4*, thereby reducing Cd translocation to the shoot.

## 3. Discussion

### 3.1. Effect of Exogenous AAs on the Growth and Photosynthetic Activity in Chinese Cabbage

Cd toxicity can inhibit root development and reduce nutrient uptake in plants, leading to chlorosis in leaves, and decrease their photosynthetic rate [33,34]. Our results suggest Met and Cys applications significantly increased shoot biomass in both cultivars and alleviated leaf chlorosis (Figure 1). Cd indirectly generates reactive oxygen species (ROS) by impairing oxidative processes or inhibiting antioxidant enzymes, thereby reducing ROS removal [35]. Consistent with previous studies [36,37], the application of Met and Cys reversed Cd-induced oxidative damage (MDA and H_2_O_2_) by increasing the activities of CAT and POD and SOD (Figure A4). Notably, supplementation with Thr or Asp did not significantly mitigate Cd toxicity in Chinese cabbage (Figure 1 and Figure 2). Therefore, we speculate that different AAs execute distinct mechanisms to alleviate Cd toxicity. The AAs that accumulate in response to Cd stress play various roles in plants [31]. For example, Cd stress tolerance is achieved in *A*. *thaliana* by the upregulation of genes associated with Cys biosynthesis [38]. Cys, a sulfur-containing AA, is involved in reduced glutathione (GSH) and phytochelatin (PC) synthesis, and regulates heavy metal homeostasis in plants [39]. GSH and PC facilitate most ABC transporters in Cd transport [40,41]. Therefore, specific transporters of these thiol ligands may alter Cd uptake in *Brassica napus* roots [42]. The application of Cys activates enzyme activity and upregulates genes associated with nitrogen metabolism, consequently improve maize seedlings tolerance to Cd stress [43].

### 3.2. Mitigation of Cd Toxicity by Modifying the Subcellular Distribution and Chemical Speciation of Cd

Several studies have indicated that exogenous AAs play a crucial role in facilitating the long-distance transportation and redistribution of Cd in plants [30,31]. Supplementation with proline and histidine effectively increases the Cd concentration in *S*. *nigrum* and *S*. *torvum* [31]. Conversely, tryptophan application vigorously inhibits Cd transport from the roots to the shoots in *Brassica oleracea* (*broccoli*) [44]. In the present study, we found that the application of Met and Cys significantly increased the Cd root-to-leaf ratio (Figure 3C). As a major precursor in the synthesis of PC [45], the application of Cys increases the Cd concentration in *Zea mays* and *B*. *napus* roots [42]. Met is a precursor in the biosynthesis of polyamines and of the phytohormone ethylene [46,47]. However, exogenous Met partly promotes the formation of Cd-Met complexes, decreasing root Cd accumulation, and enhancing Cd uptake into the shoots [46]. 

Met and Cys induced differential Cd translocation and detoxification (Figure 1 and Figure 2), which is potentially linked to the following mechanisms. The root cell wall exhibits a high Cd retention capacity, leading to reduced Cd uptake by the shoots [48]. It contains various functional groups (such as amino, carboxyl, hydroxyl, and aldehyde groups) that impede Cd mobility in plant tissues [49]. Once released from the cell wall, Cd transitions from cell wall-bound fractions to soluble fractions [50]. The soluble fraction of Cd is primarily sequestered in vacuoles, which act as a major sink and detoxification sites for Cd in both the shoots and roots [25]. Lai et al. (2015) [51] observed a positive correlation between the Cd content in shoots and the proportion of Cd in the soluble fraction of *Impatiens walleriana*. The soluble and cell wall fractions of Cd constituted a significant portion of the Cd in the shoots and roots of both cultivars (Figure 4A and Figure 6A). Our study revealed that the addition of Met and Cys significantly increased Cd concentration in the roots and cell wall fractions of both cultivars (Figure 4B and Figure 6A). The variation in Cd uptake in the shoots of both cultivars may be caused by the modulation of Cd in the cell wall fractions by the addition of Met and Cys.

Furthermore, Cd exists in the root and shoot tissues of various species and restricts its transport, thereby ameliorating Cd toxicity in plants [24]. The highest translocation was reported for inorganic and Cd-organic acid complexes, followed by pectate-integrated Cd. In contrast, Cd-phosphate, Cd-oxalate, and Cd residues exhibit the lowest translocation capacities among plants [52,53]. We demonstrated that the addition of Met and Cys improved the proportion of Cd-phosphate in the roots of Jingcui 60 and Cd-oxalate in the root of the 16-7 cultivar, respectively (Figure 5J,K). Phosphorus aggregates with Cd in the endodermal cell wall of *Azolla filiculoides*, preventing Cd translocation from the roots to the shoots [54]. Exogenous phosphorus application increases the formation of insoluble Cd phosphates, thereby reducing Cd mobility in *B*. *chinensis* [52]. Hence, we hypothesized that the Cd concentration decreased in the shoots of both cultivars under the Cd + Met and Cd + Cys treatments, which might be attributable to the increased concentration and proportion of these specific Cd specie in Chinese cabbage.

### 3.3. Alleviation of Cd Toxicity by Regulating the Expression of Metal Transporter Genes and Cd Translocation into the Xylem

Cd concentration in the xylem is regulated by the symplast and apoplast pathways, affecting the long-distance transport of Cd from the roots to shoots [55]. Cd is primarily sequestered in the cortical regions and root endodermis [56]. However, they can enter the xylem vessels and translocate to the shoot when cortical and endodermal cell wall filtration fails [57,58]. Exogenous Cys notably decreased the Cd concentration in the xylem sap of both cultivars compared to the Cd treatment (Figure A5B). We speculated that the effect of AAs on Cd reduction might be related to the transporters involved in Cd uptake. 

The PCR results show a significant increase in *IRT1* and *IRT2* expressions under Cd stress, whereas Cys and Thr applications reduce the expressions of *IRT1* and *IRT2* in Jingcui 60 (Figure 8G,H). *IRT1* transports Fe, Zn, Mn, and Cd in *pak choi* and *A*. *thaliana* [59]. Consistent with previous studies [60,61], melatonin and gibberellic acid mitigated Cd toxicity by downregulating *IRT1* expression. Plants upregulate the levels of *PCS1* and *PCS2* to promote PC formation [62]. *AtABCC1* and *AtABCC2* are responsible for transporting Cd-PC complexes for vacuolar storage [13]. However, we only observed a significant increase in *PCS1* in Jingcui 60 and *ABCC1* in the 16-7 cultivar under Cd stress (Figure 8D,E), with the Cd + Cys and Cd + Thr treatments significantly downregulating their abundance. *HMA2* and *HAM4* transporters play crucial roles in Zn and Cd loading in the xylem [17,19]. Mutations in both *hma2* and *hma4* increase sensitivity to Zn and Cd in A. *thaliana* [18]. *MYB43* decreases Cd tolerance in Arabidopsis by inhibiting *HAM2* and *HAM4* [20]. Several studies have reported that the downregulation of *HMA2* and *HMA4* reduces Cd transport in plants following the application of abscisic acid [63], gibberellin [21], and 5-Aminolevulinic acid [64]. We found that Cys application significantly downregulated the expression of *HMA2* in both cultivars and that of *HMA4* in the 16-7 cultivar (Figure 8A,B). Our results further demonstrate the potential of exogenous AA application to reduce Cd toxicity, contributing to sustainable agriculture and the quality of leafy vegetables.

## 4. Materials and Methods

### 4.1. Treatments and Conditions

We selected two varieties, the Cd-resistant cultivar Jingcui 60 and the Cd-sensitive cultivars 16-7, which were screened in our pot experiments. Under Cd-contaminated soil conditions (Cd = 1.45 mg kg^−1^, pH = 7.33), the shoot Cd concentrations in Jingcui 60 and 16-7 were 0.169 mg kg^−1^ and 0.011 mg kg^−1^, respectively. All seeds were acquired from the Jingyan Yinong Seed Science & Technology Company, Beijing, China. The seeds were transferred to seedling trays for germination after disinfection with a 4% NaClO. Briefly, the seeds were germinated on moist filter paper covered with a black plastic film at 25 °C for 48 h, after which they were transplanted into plug trays for a 2-week growth period in a climate-controlled chamber, set to 25 °C for 12 h a day with 75% relative humidity [65].

**Figure A1:**
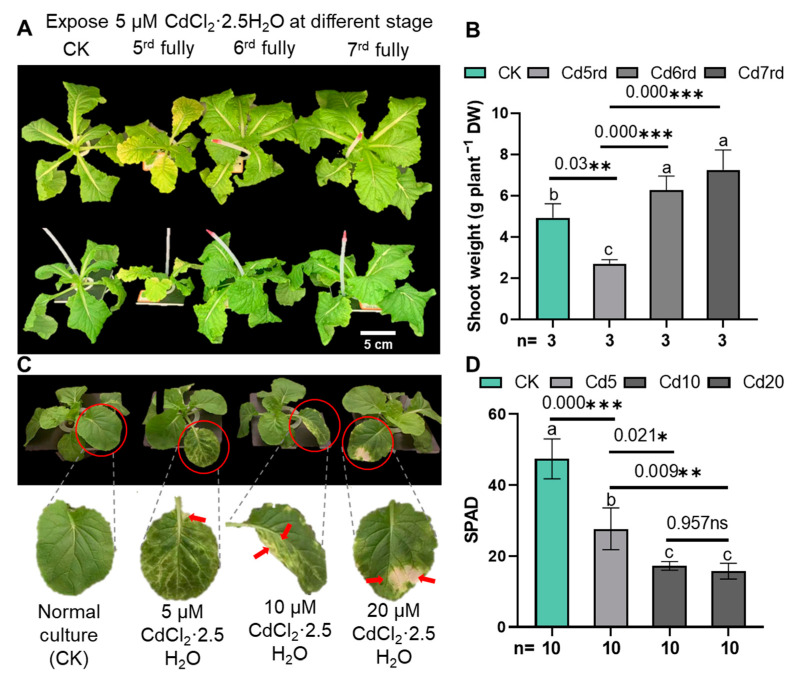
Different Cd concentrations and toxicity symptoms in Chinese cabbage. (**A**) Growth stages (5th, 6th, and 7th of full leaf) of Chinese cabbage under control conditions and treated with 5 μM Cd. (**B**) Shoot weight of Chinese cabbage under the four treatments (CK, 5th, 6th, and 7th). (**C**) Phenotypes of Chinese cabbage under control conditions and treated with 5, 10, and 20 μM Cd. (**D**) soil plant analysis development (SPAD) values of Chinese cabbage under the four treatments (CK, 5, 10, and 20 μM) in hydroponic conditions. All phenotypes were observed after four days of treatment. For all parameters, n represents the number of biologically independent samples. Different letters indicate significant differences among groups. ANOVA with Tukey’s post hoc test was used for the parametric analysis. Asterisks indicate significant differences between groups: * *p* < 0.05; ** *p* < 0.01; *** *p* < 0.001. ns indicates no significant difference.

**Figure A2:**
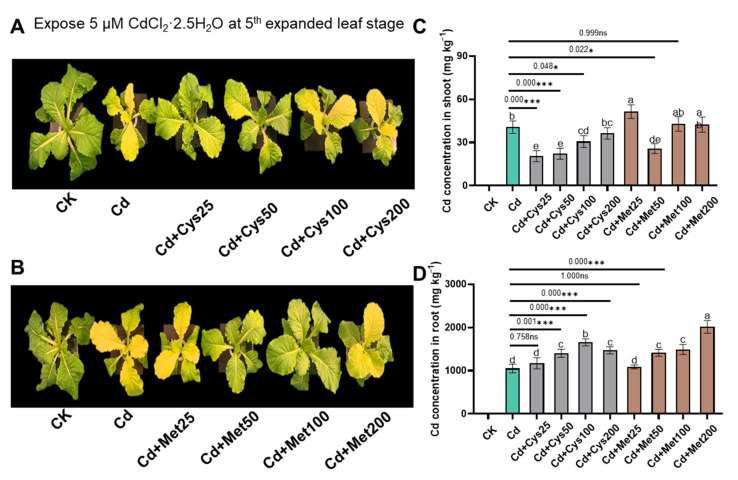
Phenotypes of Chinese cabbage (Jingcui 60) under control conditions and when treated with sole Cd and Cd + methionine (Met)/cysteine (Cys) at concentrations of 25, 50, 100, and 200 μM (**A**,**B**). The Cd concentrations in the shoots (**C**) and roots (**D**). All treatments were replicated three times. Different lowercase letters indicate significant differences among treatments. ANOVA with Tukey’s post hoc test was used for the parametric analysis. Asterisks indicate significant differences between groups: * *p* < 0.05; *** *p* < 0.001. ns indicates no significant difference.

**Figure A3:**
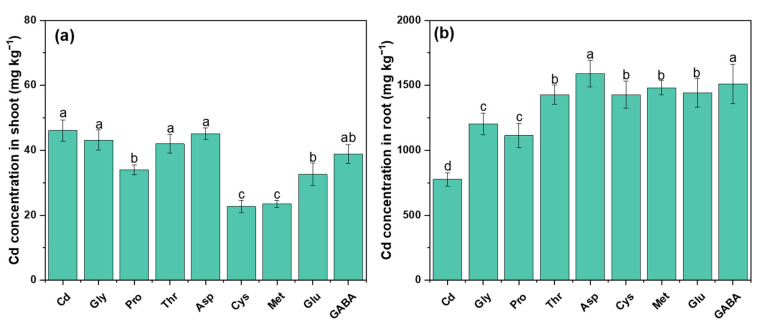
Cd concentrations in the shoots (**a**) and roots (**b**) of Jingcui 60 cultivars grown under Cd stress (5 μM) and treated with glycine (50 μM, Gly), proline (Pro), threonine (Thr), aspartic acid (Asp), cysteine (Cys), methionine (Met), glutamic acid (Glu), and gamma-aminobutyric acid (GABA). Different lowercase letters indicate significant differences among treatments. One-way ANOVA with Tukey’s post hoc test was used for the parametric analysis.

**Figure A4:**
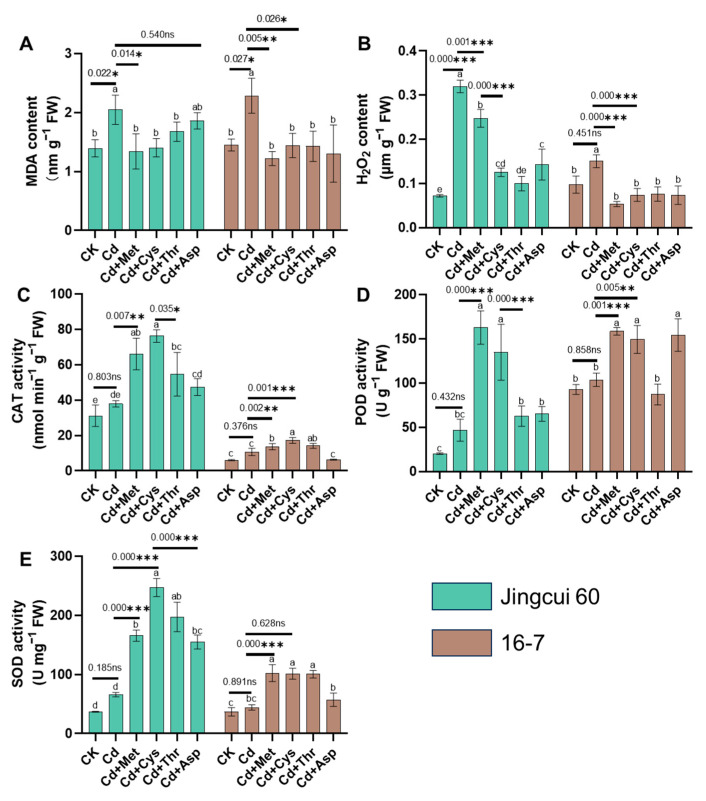
Effect of AA treatment on the activity of the antioxidant enzymes. (**A**) MDA content, (**B**) H_2_O_2_ content, (**C**) activity of CAT, (**D**) activity of POD, and (**E**) activity of SOD in the shoots. All values represent the mean ± standard deviation (SD), *n* = 3. Different lower letters indicate significant differences among treatments. ANOVA with Tukey’s post hoc test is used for the parametric test. Asterisks indicate significant differences between two groups: * *p* < 0.05; ** *p* < 0.01; *** *p* < 0.001. ns indicates no significant difference.

**Figure A5:**
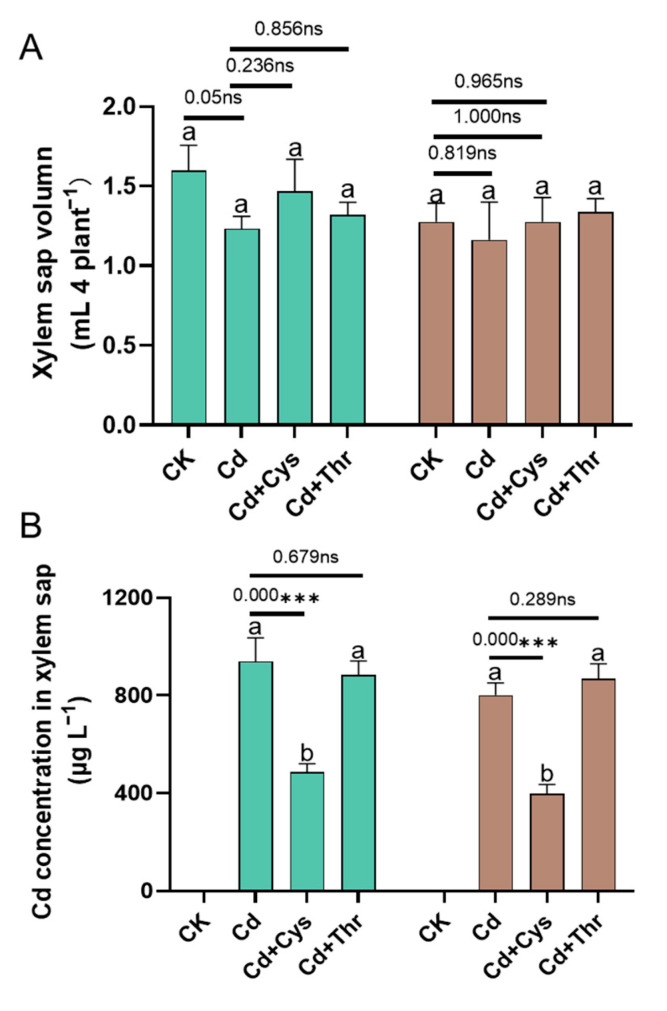
Xylem sap volumes (**A**) and Cd concentrations (**B**) of xylem sap collected from Jingcui 60 and 16-7 with Cys and Thr treatments. Data are presented as means ± SDs, and different letters on the bars indicate significant differences at α = 0.05 (n = 4 plants for each replicate). ANOVA with Tukey’s post hoc test was used for the parametric analysis. Asterisks indicate significant differences between two groups: *** *p* < 0.001. ns indicates no significant difference.

The changes in different growth stages of Chinese cabbage (fully expanded leaves at the 5th, 6th, and 7th stages) under Hoagland’s solution and 5 μM Cd treatment were observed (Figure A1A). The phenotypes of Chinese cabbage under control conditions and those treated with 5, 10, and 20 μM Cd were assessed (Figure A1C). The application level of AAs (50 μM) was determined based on our preliminary phenotypic screening at varying concentrations (25, 50, 100, and 200 μM) of AAs (Figure A2A–D and Figure A3). Each seedling was transferred to a plastic pot (height of 120 mm, top diameter of 93 mm, and bottom diameter of 63 mm), which was then filled with 400 mL of Hoagland’s solution to provide the necessary nutrients. Treatments were applied for 10 days as follows: (1) control treatment (CK); (2) Cd (5 μM CdCl_2_); (3) Cd (5 μM) + Met (50 μM); (4) Cd (5 μM) + Cys (50 μM); (5) Cd (5 μM) + Thr (50 μM); and (6) Cd (5 μM) + Asp (50 μM). All treatments commenced at the 5th fully expanded leaf stage. Each treatment was replicated three times, and each pot contained one seedling.

### 4.2. Chlorophyll Contents, Dry Weight, and Cd Accumulation Pattern

Chlorophyll contents (soil plant analysis development (SPAD)) was confirmed using SPAD-502 plus (Konica-Minolta, Tokyo, Japan), which non-destructively quantified the chlorophyll content. The SPAD values of each treatment were recorded from the 6th fully expanded true leaf prior to harvest, taking 20 measurements and averaging the results [66]. All phenotypes were observed on the fourth day of post-treatment.

Plants after ten days of these treatments. The root surfaces were cleaned of Cd by immersing them in a 20 mM EDTA-Na_2_ solution for 20 min, followed by thorough rinsing with deionized (DI) water. The samples were initially dried in an oven at 105 °C for 30 min and maintained at 75 °C until a constant weight was achieved [28]. Samples (0.2 g) were ground into a powder and digested in a microwave digester (XT-9916,Shanghai China) with 8 mL of HNO_3_ for 4 h. Subsequently, the mixtures were diluted to a fixed volume of 25 mL, filtered, and 10 mL of each sample were stored in a −4 °C refrigerator for later analysis. ICP-OES was used to determine the Cd concentration (PerkinElmer, Avio™ 200, Shelton, CT, USA) [67]. The root:shoot ratio of Cd was calculated as follows: root:shoot ratio = Cd concentration in roots/Cd concentration in shoots.

### 4.3. Various Subcellular Cd Fractionations

According to a previous study [68], the fresh tissues of leaves and roots were separated into cell walls, organelles, and soluble fractions. Briefly, fresh tissues (1 g) were ground into fine powder by adding an extraction solution (w/v = 1/10) that contained 50 mM of a Tris–HCl buffer solution (pH 7.5), 0.25 M of sucrose, and 1.0 mM of DTT (C_4_H_10_O_2_S_2_) [69]. The homogenate was centrifuged at 4000× *g* for 15 min, and the cell wall fraction was the precipitate (Hunan Xiang Yi Laboratory Instrument Development Co., Ltd., H1750R, Changsha, China). The supernatant was further centrifuged at 16,000× *g* for 30 min, and the resulting deposition and supernatant were the organelle and soluble fractions, respectively [70]. These fractions were then subjected to the digestion methods described in the previous section. Cd concentrations were determined using ICP-MS (Agilent 7900, Santa Clara, CA, USA).

### 4.4. Chemical Speciation of Cd

The chemical speciation of Cd was determined according to a previously published method [23]. In brief, 0.3 g of frozen fresh shoot and root tissues were added to 10 mL of liquid nitrogen, and the extracted solutions (w/v = 1/10) were gradually added into a mortar and then shaken for 22 h at 25 °C. The five steps of extraction were as follows: (1) immersed in 80% ethanol for inorganic Cd (F1); (2) in DI water for Cd-organic acid complexes (F2); (3) in 1 M NaCl for pectate and protein-integrated Cd (F3); (4) in 2% acetic acid for insoluble Cd-phosphate (F4); (5) in 0.6 M HCl for Cd-oxalate (F5); and (6) and Cd in residues (F6).

### 4.5. Cd Concentration in the Xylem

Xylem sap was collected as previously described, with slight modifications [71,72]. Chinese cabbages (Jingcui 60 and 16-7) were treated with 5 μM CdCl_2_, Cd + Cys (5 μM + 50 μM), and Cd + Thr (5 μM + 50 μM) for 10 days. Plants were cut 2 cm above the shoot–root junction; a 1.5 mL tube—containing pre-weighed cotton—was placed on the junction of the root and wrapped with parafilm. After 8 h, the cotton was weighed again, and the digestion process was performed in the same manner as described in the previous section. Four individual samples were randomly combined in a single tube, with twelve plants in total for each treatment.

### 4.6. Quantitative RT-PCR of Chinses Cabbage MRNA

RNA was isolated using the TRIzol reagent according to the guidelines provided by Invitrogen. RNA was transcribed into cDNA using a Verso cDNA Synthesis Kit (Thermo Fisher Scientific, Waltham, MA, USA), which incorporated a DNase I digestion stage. To measure the levels of gene expression, quantitative PCR (qPCR) was performed using specific primers for each gene (provided by BGI Genomics Co., Ltd., Shenzhen, China; refer to Appendix A Table A1 for details). The experimental design included three biological replicates for each condition, with each biological replicate comprising three technical replicates.

### 4.7. Determination of Malondialdehyde, Hydrogen Peroxide (H_2_O_2_), and Antioxidant Enzymes

The H_2_O_2_ and MDA contents of the leaves were assayed as described in our previous study [65]. The activity of antioxidant enzymes—catalase (CAT), peroxidase (POD), and superoxide dismutase (SOD)—in the leaves was measured using a reagent kit from Suzhou Comin Biotechnology Co., Ltd., Suzhou, China, as described by Ding et al. (2018) [73].

### 4.8. Statistics Analysis

Data were statistically evaluated using SPSS version 20.0. To assess the differences among the various treatments, Tukey’s test was employed, with the statistical significance set at *p* < 0.05. The relationships between the subcellular distribution and chemical forms of Cd in the roots and Cd concentrations in the shoots and roots were analyzed using PCA. Graphs were constructed using Origin 2021 and GraphPad Prism version 8.

## 5. Conclusions

In summary, our study demonstrates that Met and Cys are particularly effective in reducing Cd toxicity in Chinese cabbage. Supplementation with Met and Cys significantly decreased leaf chlorosis by increasing antioxidant enzyme activity under Cd stress. Exogenous AAs can increase the Cd concentration in the roots, but Cd-resistant varieties of Chinese cabbage fix more Cd in the cell walls, whereas Cd-sensitive varieties transport Cd to organelles. In addition, the application of Met and Cys increased the proportion of less mobile forms of Cd. Importantly, our results indicate that Cys can downregulate Cd-related transport proteins (e.g., *HMA2/4*), affecting the concentration of Cd in the xylem of Jingcui 60 and 16-7, whereas this phenomenon was not observed in the Cd + Thr treatment. Therefore, enhancing our understanding of the AAs involved in Cd detoxification in plants is crucial for developing strategies to reduce Cd accumulation in plants.

## Data Availability

Data are contained within the article.

## Appendix A

**Table A1:** A list of primer sequences used in this study.

Gene ID	cDNA Primer	Primer Forward	Primer Reverse
	Actin	5′-GGAGCTGAGAGATTCCGTTG-3’	5’-GAACCACCACTGAGGACGAT-3’
Bra009399	*HMA2*	5’-GAGGATGCCACATGGTTGGA-3’	5’-CTTTGGTACGGCGGAAGAGT-3’
Bra032640	*HMA4*	5’-TTCCCCACAAGAATCGCTCC-3’	5’-CACTCGAACCTTCCACGTCA-3’
Bra037319	*HMA3*	5’-AACCTCGACGCTATGCACAA-3’	5’-GCTTGCCACGTCATCATTGG-3’
Bra013419	*IRT1*	5’-TGGCATTCTTTTTCGCGGTG-3’	5’-GCCGAGCATGCATTGAGAAG-3’
Bra013422	*IRT2*	5’-CTCGTCGACCTTCTGGCTAC-3’	5’-ACTTGGCGACGACAGACATT-3’
Bra010773	*ABCC1*	5’-GTTGACGTTAGAACCGATGT-3’	5’-TTGAGACGATGAGCGATG-3’
Bra032385	*ABCC2*	5’-CTGTTGATGTTAGGACTGATG-3’	5’-GTGAGCGATGATGAGCAT-3’
Bra036010	*PCS1*	5’-CACAGACATGGTCAGGGAT-3’	5’-AAGCATAGTTGGGAGGGA-3’
